# Prolonged Morphine Exposure Induces Increased Firm Adhesion in an *in Vitro* Model of the Blood–Brain Barrier

**DOI:** 10.3390/ijms17060916

**Published:** 2016-06-09

**Authors:** Marianne Strazza, Vanessa Pirrone, Brian Wigdahl, Will Dampier, Wei Lin, Rui Feng, Monique E. Maubert, Babette Weksler, Ignacio A. Romero, Pierre-Olivier Couraud, Michael R. Nonnemacher

**Affiliations:** 1 Department of Microbiology and Immunology, and Center for Molecular Virology and Translational Neuroscience, Institute for Molecular Medicine and Infectious Disease, Drexel University College of Medicine, Philadelphia, PA 19102, USA; ms996@drexel.edu (M.S.); vpirrone@drexelmed.edu (V.P.); bwigdahl@drexelmed.edu (B.W.); wnd22@drexel.edu (W.D.); mem446@drexel.edu (M.E.M.); 2 Sidney Kimmel Cancer Center, Thomas Jefferson University, Philadelphia, PA 19107, USA; 3 School of Mathematical Sciences and Center for Statistical Science, Peking University, Beijing 100871, China; weilin@math.pku.edu.cn; 4 Department of Biostatistics and Epidemiology, Center for Clinical Epidemiology and Biostatistics, University of Pennsylvania School of Medicine, Philadelphia, PA 19104, USA; ruifeng@upenn.edu; 5 Department of Medicine, Division of Hematology and Oncology, Weill Cornell Medical College, New York, NY 10065, USA; babette@med.cornell.edu; 6 Department of Biological Sciences, Open University, Walton Hall, Milton Keynes MK7 6AA, UK; i.romero@open.ac.uk; 7 Institut Cochin, INSERM (Institut National de la Santé et de la Recherche Médicale), U1016, CNRS UMR8104, Université Paris Descartes, Sorbonne Paris Cité, Paris 75014, France; pierre-olivier.couraud@inserm.fr

**Keywords:** blood–brain barrier, brain microvascular endothelial cells (BMEC), morphine, (peripheral blood mononuclear cell) PBMC, cellular adhesion molecules (CAM)

## Abstract

The blood–brain barrier (BBB) has been defined as a critically important protective barrier that is involved in providing essential biologic, physiologic, and immunologic separation between the central nervous system (CNS) and the periphery. Insults to the BBB can cause overall barrier damage or deregulation of the careful homeostasis maintained between the periphery and the CNS. These insults can, therefore, yield numerous phenotypes including increased overall permeability, interendothelial gap formation, alterations in cytokine and chemokine secretion, and accelerated cellular passage. The current studies expose the human brain microvascular endothelial cell line, hCMEC/D3, to prolonged morphine exposure and aim to uncover the mechanisms underlying alterations in barrier function *in vitro*. These studies show alterations in the mRNA and protein levels of the cellular adhesion molecules (CAMs) intercellular adhesion molecule-1, vascular cell adhesion molecule-1, and activated leukocyte cell adhesion molecule that correlate with an increased firm adhesion of the CD3^+^ subpopulation of peripheral blood mononuclear cells (PBMCs). Overall, these studies suggest that prolonged morphine exposure may result in increased cell migration into the CNS, which may accelerate pathological processes in many diseases that involve the BBB.

## 1. Introduction

The endothelium of the blood–brain barrier (BBB) is composed of brain microvascular endothelial cells (BMECs), a distinct form of endothelial cells [1]. Underlying the BMECs is the basal lamina, followed by other cell types, including astrocytes, pericytes, perivascular macrophages, parenchymal microglia, and neurons, which are all involved in maintaining the microenvironment that preserves the functionality of BMECs [1,2,3,4,5]. BMECs form an unfenestrated endothelium, which, in combination with intervening tight junction complexes (TJC) and the low number of intracellular vesicles under normal conditions, causes the BBB to have selective permeability to peripheral cells, proteins, and other molecules [6,7,8].

TJC are made up of multiple proteins including the transmembrane proteins of the claudin family and occludin, as well as the intracellular accessory protein zonula occludin 1 (ZO-1). The link to the actin cytoskeleton through ZO-1 is essential for proper TJC formation and function [9], and loss or relocalization of this protein disrupts BBB function. Movement of circulating lymphocytes into tissues also involves interaction between the transmigrating cells and the endothelium of post-capillary venules, an interaction that is mediated through cellular adhesion molecules (CAMs) on the surface of both cell populations. Changes in surface expression of CAMs on either cell population in response to activating stimuli control movement of lymphocytes into the tissue [10,11,12,13,14]. Basal immune surveillance of the central nervous system (CNS) under normal, non-inflammatory conditions is greatly reduced compared with that in other tissues, such as the lungs and spleen, owing to the limited expression of CAMs on BMECs [15,16]. In light of this, any change in expression of CAMs can have a major effect on proper barrier function and immune cell access and surveillance.

Treatment of the BBB with morphine for 24 h has been shown to decrease ZO-1 and occludin gene expression, and increase transmigration of peripheral blood mononuclear cells (PBMCs) [17] without having a large influence on tracer molecule passage [18,19]. The nature of the functional studies impacted by morphine previously observed has suggested that alteration in tight junction protein (TJP) expression is not solely responsible for mediating the changes in PBMC transmigration. In the current studies, we set out to identify the distinct mechanism through which prolonged morphine exposure may alter cellular passage across the BBB while not inducing non-specific leakiness.

The human BMEC cell line, hCMEC/D3, was utilized in an *in vitro* model of the BBB to address the effects of morphine on the barrier. The cell line was established through the isolation of microvessels from the temporal lobe [20]. The stability of this cell line for up to six days post-confluence [20,21,22,23,24,25] allowed us to study the effects of up to 72 h of drug treatment on BMECs, *in vitro*. In addition, the hCMEC/D3 cell line maintains BMEC characteristics in the absence of co-culture with astrocytes, pericytes, or neurons that are often employed in other BBB models [20,21,22,23]. Through the use of the hCMEC/D3 monolayer alone, these studies directly assess the impact of morphine on BMECs alone without the confounding effects of a second or even third cell type. Therefore, the use of this well established single cell model in these studies allows for the exploration of the basic pathologic mechanisms associated with morphine exposure that can be expanded in future studies to include the secondary impact involving co-culture with other cell types. In these studies, we have shown that prolonged exposure to morphine altered mRNA and protein expression of CAMs within BMECs and increased firm adhesion of PBMCs to the endothelium.

## 2. Results

### 2.1. Morphine Does Not Induce BBB (Blood–Brain Barrier) Leakiness

To address the effects of prolonged exposure to morphine in an *in vitro* BBB model required a model that maintained functional confluence longer than 24 h beyond the point at which confluence was first reached. The studies reported here extend morphine treatment to 48 and 72 h, bringing the total time beyond confluence to 96 h. Because of the nature of these studies, the hCMEC/D3 human BMEC cell line was ideally suited for use in this line of experimentation. The human BMECs have been immortalized using human telomerase reverse transcriptase and simian vacuolating virus 40 large T antigen [20] and have been shown to maintain functional characteristics comparable to primary human BMECs for up to seven days following establishment of confluence [21,22]. In addition, the ability of hCMEC/D3 cells to maintain function in the absence of astrocyte co-culture greatly facilitated studies to analyze the direct effects of morphine on the endothelial cell population without any secondary indirect effects that may be mediated by other cell populations in co-culture.

Previous *in vitro* studies involving morphine and BBB function have examined the impact of 24 h exposure on tracer molecule passage across the monolayer, TJP expression, and PBMC transmigration. While many of these studies concluded that morphine does not increase BBB permeability through tracer molecule passage [18,19], others have shown alteration in TJP expression and accelerated PBMC transmigration [17].

**Figure 1:**
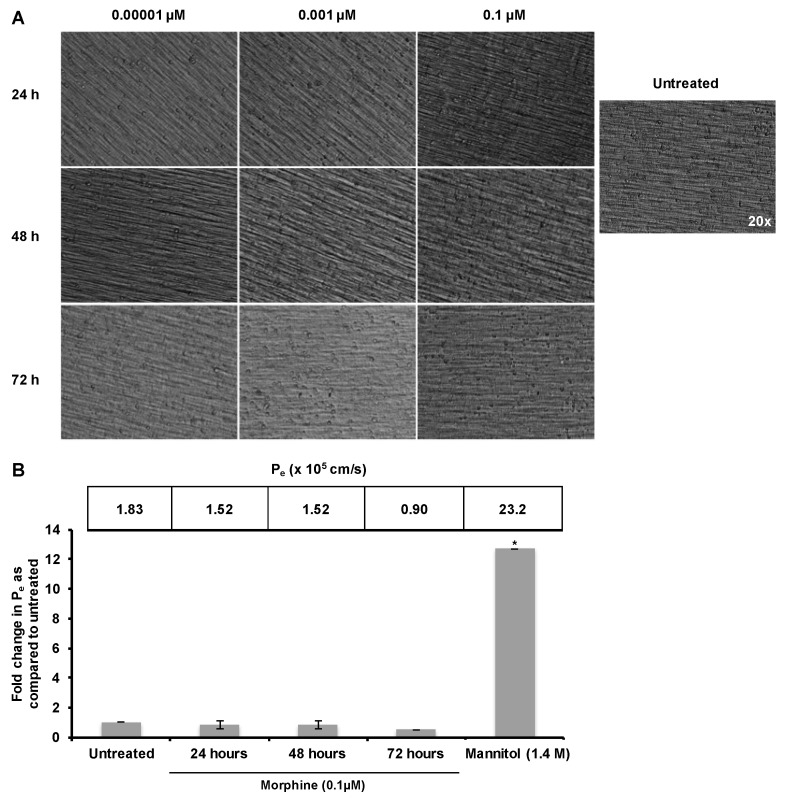
Morphine did not induce leakiness of the hCMEC/D3 barrier. (**A**) hCMEC/D3 cells were cultured for 10 days on 0.4 µM porous polytetrafluoroethylene (PTFE) transwell inserts. The confluent monolayer was then exposed to morphine (0.1 µM) for 24, 48, or 72 h with morphine additions every 24 h to maintain drug concentration. No changes in cellular morphology were observed. Monolayers incubated in the absence or presence of morphine were visualized under 20× Relief Contrast Microscopy using an Olympus (Center Valley, PA, USA) IX81 deconvolution microscope; (**B**) hCMEC/D3 cells were cultured and treated as in panel A. Permeability was assessed by determining the amount of 70 kDa fluorescein isothiocyanate–dextran (FITC-D) to pass from the upper to lower chamber over 30 min, and the permeability coefficient (*P_e_*) was calculated. Results show that treatment with morphine does not cause general, non-specific leakiness of the monolayer. All treatments were performed in triplicate and are representative of three independent experiments. Statistical analysis was performed using the analysis of variance (ANOVA) method with log transformation and adjustment for possible time effects to compare the results obtained with untreated monolayers to the results obtained with the morphine-treated monolayers and to the results obtained with monolayers treated with mannitol. Based on 95% confidence intervals, no significant change was observed with morphine treatment. * *p* < 0.0001.

The impact of a substance on BBB structure and function can be induction of widespread leakiness and enhanced permeability or activation of the endothelium leading to disrupted regulation of passage. Based on these previous results, it was hypothesized that prolonged morphine exposure would activate BMECs, leading to increased CAM expression and increased PBMC firm adhesion to the endothelium. To distinguish between these two possibilities of nonspecific leakiness and endothelial activation, hCMEC/D3 cells were exposed to morphine (0.001, 0.01, or 0.1 µM) for 24, 48, or 72 h with re-administration at 24 h intervals. These concentrations of morphine were selected both based on usage in previous *in vitro* and *in vivo* studies, and due to the fact that they fall within the clinically observed serum concentration range of patients receiving intravenous morphine [17,26,27]. Morphine was not observed to induce any morphological changes in the cell monolayer, such as cell rounding or gap formation (Figure 1A). A fluorescein isothiocyanate–dextran (FITC-D) permeability assay was then performed in order to quantitate the rate of small molecule passage across the endothelium following treatment with morphine (0.1 µM). Treatment of the confluent monolayer with mannitol (1.4 M), a compound commonly used clinically to enhance transport of therapeutics across the BBB, induced a significant increase in *P_e_* (Figure 1B), indicating that the low *P_e_* observed did not result from cell piling and a physical blockade of the insert pores. Based on the FITC-D permeability assay, prolonged morphine exposure did not induce a significant increase in *P_e_* when compared with untreated monolayers (Figure 1B). These results suggest that morphine does not induce general, non-specific leakiness of the hCMEC/D3 monolayer, and therefore, may specifically enhance the permissiveness to cellular transmigration through an alternate mechanism.

### 2.2. Morphine Alters CAM (Cellular Adhesion Molecule) mRNA and Protein Expression

The cellular adhesion molecules ICAM-1, VCAM-1, and ALCAM have been shown to be regulated independently and have differing kinetics following endothelial cell activation [28,29]. Collectively, CAMs play an essential role in cellular transmigration and tissue extravasation, through mediating the interaction between the endothelial cells and the extravasating cell. Surface expression levels of CAMs are increased upon cellular stimulation, including inflammatory signaling [30,31,32,33], HIV-1 proteins [34,35], and cocaine exposure [36]. To determine the effects of prolonged morphine exposure on CAM expression, mRNA levels of ICAM-1, VCAM-1, and ALCAM were examined following exposure.

**Figure 2:**
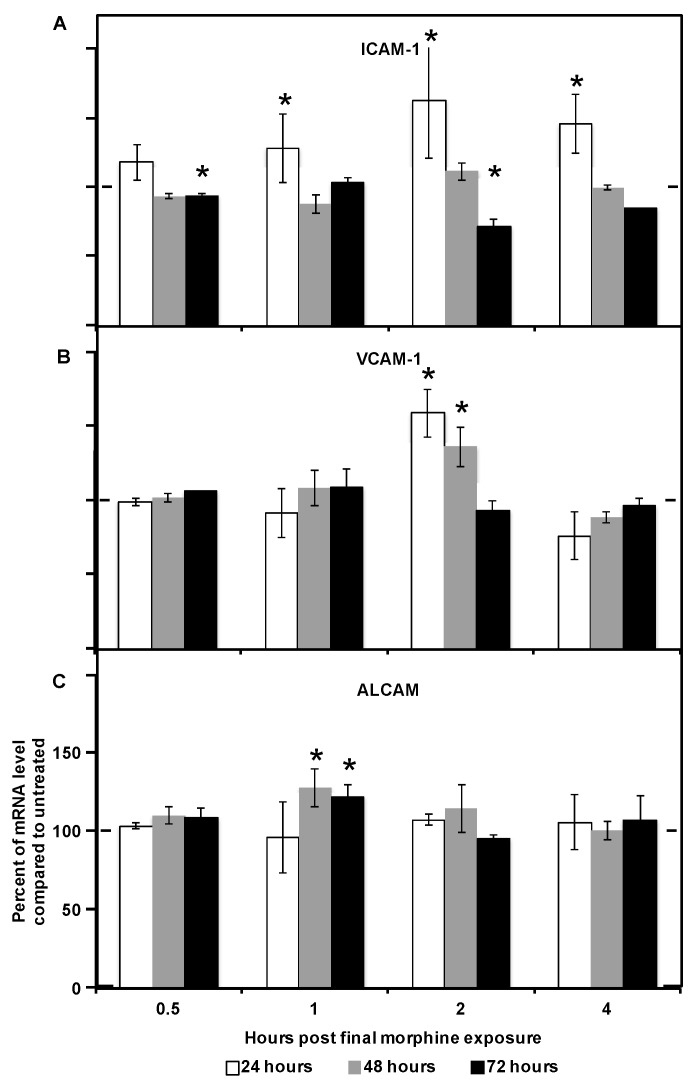
Prolonged morphine exposure induced changes in mRNA levels of CAMs (cellular adhesion molecules) in hCMEC/D3 cells. hCMEC/D3 cells were treated with morphine as previously described. Intercellular adhesion molecule-1 (ICAM-1; **A**), vascular cell adhesion molecule-1 (VCAM-1; **B**), and activated leukocyte cell adhesion molecule (ALCAM; **C**) mRNA levels were measured by RT-PCR at the indicated time points with total exposure times of 24, 48, or 72 h. All bars represent average percent of relative mRNA level compared with the untreated condition, with the values obtained from untreated monolayers normalized to 100%. Averages were derived from two independent experiments performed in duplicate. Relative mRNA was calculated based on the geometric mean of β-actin and glyceraldehyde 3-phosphate dehydrogenase (GAPDH). Levels of β-actin and GAPDH did not change as a result of any treatments. * *p* < 0.05 as determined by Student’s *t*-test. The dashed line is set to 100% for ease of comparison.

Following 24 h of continuous morphine exposure, ICAM-1 and VCAM-1 mRNA levels were significantly increased as compared to their expression levels in untreated cells (Figure 2A,B). ICAM-1 mRNA was increased by 1 h following the final re-administration of morphine and the levels remained elevated for up to 4 h following the final re-administration. Levels of VCAM-1 mRNA were increased within 2 h following the final re-administration of morphine and returned to baseline by 4 h. Levels of ALCAM mRNA were not altered following 24 h of continuous morphine exposure (Figure 2C).

At the conclusion of 48 h of continuous morphine exposure, VCAM-1 and ALCAM mRNA levels were significantly increased (Figure 2B,C). The kinetics of the observed VCAM-1 increase matched the kinetics observed following 24 h of exposure. ALCAM mRNA levels increased within 1 h of the final re-administration of morphine and returned to baseline by 4 h following re-administration. Interestingly, ICAM-1 mRNA levels were unchanged following 48 h of morphine exposure (Figure 2A).

Following 72 h of continuous morphine exposure, ICAM-1 significantly, though intermittently, decreased (Figure 2A). Cells that had been in the presence of morphine for 72 h had decreased ICAM-1 mRNA levels by 30 min after the final addition of morphine; levels returned to baseline by 1 h after the final addition and decreased again by 2 h following the final re-administration of morphine. In addition, ALCAM mRNA levels were significantly increased following 72 h of morphine exposure, with kinetics that correlated to those observed following 48 h of exposure (Figure 2C). No significant change in VCAM-1 mRNA was observed as a result of 72 h of continuous morphine exposure (Figure 2B). These results suggest that distinct signaling pathways may be involved in response to 24, 48, and 72 h of morphine exposure in hCMEC/D3 cells. These morphine exposure kinetic profiles may be the result of changes in signaling as the total exposure time increases, or they may result from feedback involving the protein expression directed by these genes.

**Figure 3:**
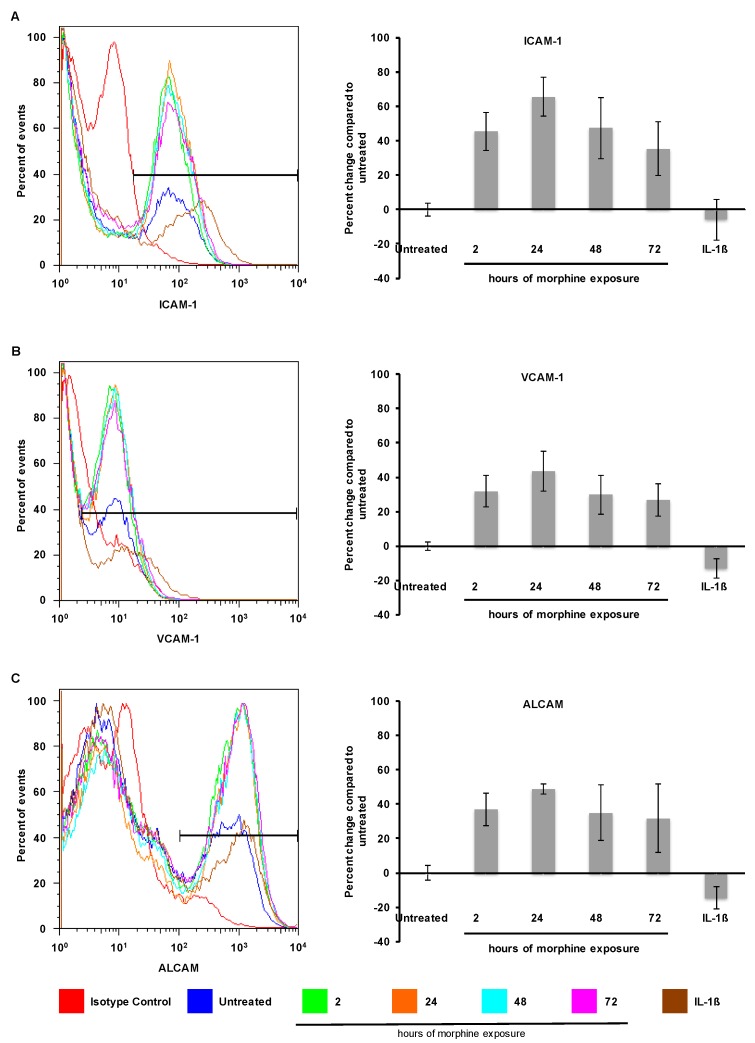
Morphine increased cell surface expression of intercellular adhesion molecule-1 (ICAM-1), vascular cell adhesion molecule-1 (VCAM-1), and activated leukocyte cell adhesion molecule (ALCAM). Exposure to morphine (0.1 µM) for 2, 24, 48, and 72 h increased the number of hCMEC/D3 cells expressing ICAM-1 (**A**); VCAM-1 (**B**); and ALCAM (**C**) on the cell surface as determined by flow cytometry on nonpermeabilized hCMEC/D3 cells (panels on left). The levels of expression in untreated cells are shown in blue; individual isotype controls are shown in red for each antibody treatment. A range gate, as shown in these histograms, was used to quantitate the number of cells within a given fluorescence intensity (FI) range. Treatment with IL-1β (20 ng/mL) for 24 h induced an increased level of ICAM-1, VCAM-1, and ALCAM expression (compare the blue and brown lines in the left panels), while it slightly decreased the number of cells expressing the proteins on the cell surface (right panels). Quantitation of hCMEC/D3 cells expressing ICAM-1 (**A**); VCAM-1 (**B**); and ALCAM (**C**) is shown as the percent change in the number of expressing cells as compared with untreated controls (panels on right). The untreated control is shown as 0% change. All bars represent average percent change in the number of cells compared with the untreated control. All bars represent three independent experiments performed in triplicate. *p* < 0.001 for all treatment conditions compared with untreated controls by Student’s *t*-test.

**Figure 4:**
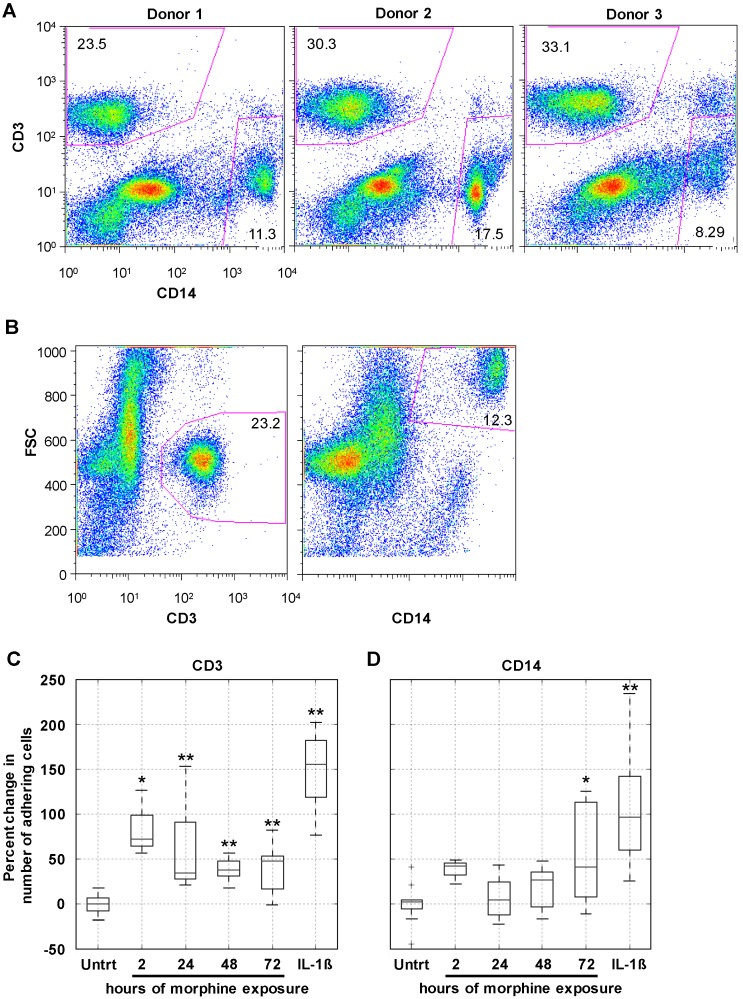
Prolonged morphine exposure increased peripheral blood mononuclear cell (PBMC) firm adhesion. hCMEC/D3 cells were grown to confluence as described earlier. At confluence, cells were exposed continuously to morphine (0.1 µM) for 2, 24, 48, or 72 h with re-administration at 24 h intervals. The final morphine administration preceded the adhesion assay by 2 h. As a positive control of enhanced adhesion, confluent hCMEC/D3 cells were exposed to IL-1β (20 ng/mL) for 24 h. Following treatment, a suspension of 1 million PBMCs in co-culture media was added to the hCMEC/D3 monolayer and the co-cultures were incubated at 37 °C in 5% CO_2_ for 30 min to allow adhesion to occur. (**A**) At the start of each adhesion assay, the start population of PBMCs was stained for CD3 and CD14 and analyzed by flow cytometry. Each donor had comparable CD3^+^ and CD14^+^ viable cell populations, indicating that the representation of these cell types in the initial population was not the driving force behind any observed differences in selective cell adhesion; (**B**) population gates were established on forward scatter (FSC) and either CD3 or CD14, and the number of events within each of these gates was used in the analysis as the number of CD3^+^ and CD14^+^ PBMCs that adhered to the hCMEC/D3 monolayer. Representative scatter plots from Donor 1 are shown here; (**C**) morphine treatment for 2, 24, 48, and 72 h, as well as IL-1β treatment of the monolayer, increased the number of CD3^+^ cells firmly adhering to the hCMEC/D3 monolayer, as quantitated by CD3 staining and flow cytometric analysis; (**D**) morphine treatment of the monolayer significantly induce firm adhesion of CD14^+^ cells but only at the 72 h time point. Treatment of the monolayer with IL-1β is shown as a positive control for increased adhesion. All bars represent three independent experiments performed in triplicate, each with individual PBMC donor samples. * *p* ≤ 0.02; ** *p* ≤0.002 as determined by Student’s *t*-test.

The functional impact of the observed RNA changes was assessed by identifying accompanying changes at the protein level through cell surface staining for CAMs to quantitate the levels of surface expression. To determine whether surface expression of ICAM-1, VCAM-1, or ALCAM was increased by morphine treatment, confluent hCMEC/D3 monolayers were treated with morphine (0.1 µM) for 2, 24, 48, or 72 h and surface expression was analyzed through flow cytometry of nonpermeabilized cells. Interestingly, 2, 24, 48, and 72 h of morphine treatment all significantly increased the number of hCMEC/D3 cells expressing ICAM-1, VCAM-1, and ALCAM (Figure 3); this observation was in contrast to the increased expression level on already expressing cells (compare the blue and brown lines, Figure 4, left panels) with a slight decrease in the number of cells expressing these molecules (Figure 3, right panels) that was shown after treatment with IL-1β (20 ng/mL) for 24 h. Surface expression of these adhesion molecules has the potential to increase interaction between PBMCs and BMECs and, therefore, to increase cellular passage.

### 2.3. Morphine Increases PBMC Adhesion

A critical function of the BBB centers on the regulation of cellular passage from peripheral circulation into the CNS. In order for PBMCs to gain entry into the CNS, an interaction involving reciprocal activation of the PBMCs and the endothelial cells is required [37]. To assess the impact of prolonged morphine exposure on the interaction between PBMCs and the hCMEC/D3 BBB model, adhesion of PBMCs was quantitated. As mentioned, PBMC transmigration across the BBB involves a highly regulated interaction between the PBMCs and the endothelial cells, involving activation of both cell types. Therefore, an increase in cellular passage following an insult to the BBB may be the result of widespread leakiness and unregulated passage or enhanced cellular activation and deregulated passage. Given that no increase in FITC-D passage was observed as a result of morphine treatment (Figure 1B), the number of CD3^+^ (T-cell) and CD14^+^ (monocyte) cells firmly adhering to the hCMEC/D3 monolayer was assessed following morphine exposure.

At the conclusion of morphine exposure of the hCMEC/D3 monolayer in a flat bottom plate, primary human PBMCs in a suspension of co-culture media were added and allowed to incubate for 30 min at 37 °C in CO_2_ (5%) to allow firm adhesion to occur. A sample cell population from all donors for CD3 and CD14 was analyzed to ensure that each donor included in the assays had a normal presence of both single-positive populations (Figure 4A). Following the 30-min incubation, any unbound cells were removed through washing, and all remaining cells were collected and stained for CD3 and CD14. Single-positive populations for CD3 and CD14 were included based on forward scatter to eliminate any hCMEC/D3 cells from the population being quantitated (Figure 4B).

When compared with the level of adhesion to untreated monolayers (set at 0%), 24, 48, and 72 h of morphine treatment significantly increased the level of firm adhesion of CD3^+^ cells (Figure 4C). Activation of the hCMEC/D3 cells with IL-1β (20 ng/mL) for 24 h significantly increased the level of CD3^+^ cell firm adhesion, as expected, and was included in these assays as a positive control. Interestingly, only the 72-h morphine treatment enhanced firm adhesion of the CD14^+^ population of cells (Figure 4D). While the variability in the percentage of the overall starting CD14^+^ population was greater than that of the CD3^+^ population (Figure 4A), IL-1β treatment still consistently and significantly increased CD14^+^ cell firm adhesion (Figure 4D).

To confirm the correlation between increased surface expression of ICAM-1, VCAM-1, and ALCAM with increased CD3^+^ firm adhesion to a morphine-treated monolayer, a linear regression analysis was performed. The number of adhered CD3^+^ cells was plotted against either the number of expressing cells or the mean fluorescence intensity (MFI) obtained with ICAM-1, VCAM-1, or ALCAM. This analysis was performed using the averages obtained from the three independent experiments performed in triplicate presented in Figure 3 and Figure 4. An increase in the number of cells expressing ICAM-1, VCAM-1, and ALCAM correlated with an increase in the number of adhered CD3^+^ cells (Table 1). Interestingly, the small increase in MFI of ICAM-1 and ALCAM also correlated with an increase in CD3^+^ cell adhesion. This analysis further suggested a correlation between the observed increase in surface expression of ICAM-1, VCAM-1, and ALCAM with CD3^+^ cell adhesion. The same analysis was performed using the number of adhered CD14^+^ cells, and no correlation was identified (data not shown).

## 3. Discussion

In the studies presented here, the hCMEC/D3 cell line was used because it has been demonstrated to be stable in monoculture for several days after reaching confluence, allowing us to study the direct effects of prolonged drug exposure on BMEC biology. Previous studies of the effects of morphine on the BBB used various *in vivo* rodent models as well as *in vitro* primary BMEC models either in co-culture with other cell types, such as astrocytes, or as a monoculture. While *in vivo* rodent models make long-term drug studies possible, issues arise with differences in drug metabolism. Primary BMEC *in vitro* models, particularly co-cultures with primary astrocytes, closely model the physiological characteristics of the human BBB *in vivo*. However, these models are not well suited for studies extending beyond 24 h post-confluence and for this reason are problematic for prolonged drug exposure studies. In addition, the study of an endothelial monolayer allowed for analysis of the direct impact of morphine on endothelial cell biology. In order to identify changes in BBB function mediated by other cell types of the BBB, co-culture can be incorporated into the hCMEC/D3 model in future studies.

We have found that prolonged morphine exposure, beyond 24 h, causes an increase in the firm adhesion of a CD3^+^ subpopulation of PBMCs. These functional results correlate with increased mRNA levels and surface expression of ICAM-1, VCAM-1, and ALCAM. Upregulation of CAMs indicated an inflammatory response and suggested an altered cytokine or chemokine expression profile, a possibility that should be further explored. While these studies confirm earlier results addressing the effects of morphine exposure [17,18,19], these studies are the first such observations to address the distinction between gross BBB disruption and increased interaction with PBMCs. These results highlight the importance of studying BMEC biology beyond TJP regulation, which has been shown to be altered following short-term morphine exposure [17], since TJ disruption is only one possible mechanism through which BBB function may be altered.

Recently, the binding of morphine to TLR-4 has been characterized and the results suggested that signaling through TLR-4 mediates the inflammation that often accompanies morphine administration *in vivo*, either in an abusive or clinical context [38]. Highlighting the complexities and interplay of these morphine-mediated pathways in the context of pathogenesis, a role for *in vivo* subcutaneous morphine exposure in the upregulated expression of TLR-4 on monocytes isolated from murine prefrontal cortices, paired with the CNS infiltration of bacteria-infected monocytes by a mu-opioid receptor (MOR)-mediated mechanism, has also recently been reported [39]. Significant ongoing research on opioids is thus aimed at formulating a “better pain reliever” that will separate the analgesic properties of opioids from the immune-modulating side effects, particularly neuroinflammation [38,40]. These results leave open the possibility that signaling through TLR-4 in BMECs comprising the BBB may contribute to the development of neuroinflammation in humans as a result of morphine treatment. Indeed, it has been demonstrated that primary rat CNS endothelial cells do express functional TLR-4, which is stimulated in the presence of both morphine and morphine-3-glucuronide, a metabolic derivative of morphine, and gives rise to a pro-inflammatory state, both *in vitro* and *in vivo* [41]. Recent contradicting reports on the potential role of *in vitro* exposure to lipopolysaccharide (LPS) in the induction of CAM expression in murine brain endothelial cells further emphasizes the need for additional investigation into the role of morphine-mediated activation of both MOR and TLR-4 pathways in human-based systems [42,43]. The possibility that the observed increase in CAM protein expression and PBMC firm adhesion reported herein may be mediated through either MOR-1 or TLR-4 signaling has many implications for future studies of morphine, not only in the context of abuse, but also in a clinical context.

The observation that morphine specifically increased firm adhesion of CD3^+^ T cells was surprising and has implications for the downstream, long-term effects of morphine on the BBB from a physiological point of view. Immune surveillance of the normal CNS is mediated primarily by CD3^+^ cells [44]. Transmigration of the CD3^+^ population across an unstimulated BBB was dependent on ICAM-1 expression, whereas migration of the same cell population following IL-1β stimulation of the BBB was dependent on both ICAM-1 and VCAM-1 [45]. These observations correlate with the studies reported herein; with the largest increase in expression observed being ICAM-1, and this increase was accompanied by a selective increase in CD3^+^ cell adhesion. Additionally, IL-1β treatment increased expression of both ICAM-1 and VCAM-1 to nearly equal levels and in turn increased firm adhesion of both the CD3^+^ and CD14^+^ populations. Overall, these studies have implications for a number of diseases including neuroinflammatory syndromes, as well as for understanding HIV-1 neuropathogenesis, as intravenous drugs such as heroin and morphine are widely abused by patients with HIV-1 infection. A link between T-cell passage into the CNS and HIV-1 neuropathogenesis has been suggested through viral models of both early disease [46] and after the onset of neurological disorders [47]. Based on these observations, additional studies characterizing the CD3^+^ subpopulation that has been observed to firmly adhere will be important in further elucidating the effects of morphine on the BBB. Additional studies should also be aimed at better understanding the mechanism mediating these changes.

## 4. Experimental Section

### 4.1. Reagents

Morphine sulfate, mannitol, and FITC-dextran (FITC-D) (70 kDa), were obtained from Sigma Aldrich (St. Louis, MO, USA). Stromal cell–derived factor-1 (SDF-1α) was obtained from R&D Systems (Minneapolis, MN, USA). IL-2 was obtained from the NIH AIDS Reagent Program (Germantown, MD, USA).

### 4.2. Cell Culture and Treatment

Cells from the human BMEC line hCMEC/D3 (provided by Dr. Babette Weksler) were cultured in endothelial basal medium-2 supplemented with heat-inactivated fetal bovine serum (FBS) Gold (5%), penicillin-streptomycin (1%), hydrocortisone (1.4 µM), ascorbic acid (5 µg/mL), chemically defined lipid concentrate (1%), HEPES (10 mM), and bFGF (1 ng/mL) (referred to herein as CMEC media). All experiments were conducted between passages 27 to 32. Cells were grown on Petri dishes, 6-, 12-, or 24-well plates coated with Cultrex rat collagen I (Trevigen, Gaithersburg, MD, USA) (150 μg/mL) in H_2_O. hCMEC/D3 cells were seeded on Petri dishes (6-, 12-, and 24-well plates) at a density of 37,000 cells/cm^2^ or on transwell inserts (details below). At confluence on plates or in transwells, hCMEC/D3 cells were treated where indicated with morphine (0.001, 0.01, or 0.1 µM), concentrations previously reported [17] for 2, 24, 48, or 72 h; with mannitol (1.4 M) for 30 min; or with IL-1β (30 ng/mL). These concentrations were used because 100 nM of morphine is the mid-range concentration of intranasal *versus* intravenous plasma levels and because this is the most common concentration cited in the literature. This was calculated from heroin (5 mg) intravenous results in a peak plasma morphine concentration of 0.035 mg/L or 120 nM. If one injects 12 mg of heroin intravenously, this results in a peak plasma morphine concentration of 0.044 mg/L or 120 nM. Doses of IV heroin of 150 to 200 mg result in plasma morphine concentrations of up to 0.3 mg/L or 1.05 μM. Intranasal administration of 12 mg heroin results in 0.019 mg/L or 70 nM of morphine at 0.08–1.5 h. Treatments for each individual experiment were coordinated such that all cells remained in culture for an equivalent amount of time. For prolonged morphine exposures, morphine was re-administered every 24 h throughout the course of prolonged treatment in order to maintain consistent levels of drug in the culture system.

**Figure 5:**
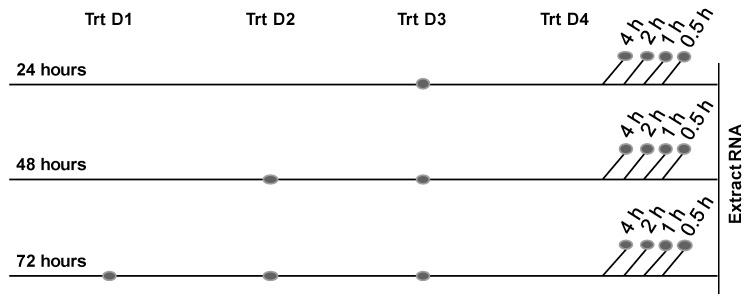
Protocol for morphine treatment. At confluence, hCMEC/D3 cells were incubated in the absence or presence of morphine (0.1 µM) for 24, 48, or 72 h (Trt D1, Trt D2, *etc.*), during which time morphine was re-administered every 24 h to maintain drug concentration. Untreated cells received an equivalent volume of media. At the indicated time points after the final addition of morphine, RNA was extracted. The dots indicate the points in time that morphine was added to the culture. The 24, 48, and 72 h indicate the total time of morphine exposure, and the noted time increments indicate the duration elapsed between the final morphine addition and RNA extraction.

For RNA studies, morphine was administered on a staggered schedule (0.5, 1, 2, or 4 h) prior to RNA extraction; this procedure allowed the total time of morphine exposure to remain 24, 48, or 72 h with variation only in the elapsed time since the final morphine administration (Figure 5), which was crucial to the observation of rapid changes in RNA expression. Prior to protein analysis, or the adhesion assay, administration of morphine was staggered as described above, with the final administration of drug 2 h prior to harvest.

Primary human PBMCs were obtained from the Temple University School of Medicine Comprehensive NeuroAIDS Center (Philadelphia, PA, USA) (Basic Science Core 1) and cultured at 1 million cells/mL in Roswell Park Memorial Institute (RPMI) supplemented with heat-inactivated FBS (10%) and penicillin-streptomycin (1%) in the presence of IL-2 (0.1 ng/mL) (referred to herein as PBMC media) in polypropylene tubes to discourage monocyte differentiation and adherence.

For assays involving co-culture of PBMCs and hCMEC/D3 cells, a distinct media was formulated with RPMI supplemented with heat-inactivated FBS (10%), penicillin-streptomycin (1%), ascorbic acid (5 µg/mL), chemically defined lipid concentrate (1%), HEPES (10 mM), and bFGF (1 ng/mL) and referred to herein as co-culture media.

### 4.3. In Vitro Blood–Brain Barrier Model

hCMEC/D3 cells were seeded on collagen-coated porous polytetrafluoroethylene (PTFE) transwell inserts (Corning, Inc., Corning, NY, USA; 0.4 µm) at a density of 4.5 × 10^4^ cells/cm^2^ and grown for 10 days to achieve confluence for use in FITC-D permeability assays.

### 4.4. FITC-Dextran Permeability Assay

Indicated treatments of the hCMEC/D3 monolayers were performed at confluence on porous PTFE transwell inserts (0.4 µm). The monolayers were then washed with 10 mM HEPES in 1× Hanks’ balanced salt solution (HBSS), and 70-kDa FITC-D (2 mg/mL) in CMEC media was added to the upper chamber. Passage through the monolayer was monitored through sampling from the lower chamber at 5 min intervals over 30 min (6 time points); these samples were transferred to 96-well optical bottom plates and read for fluorescence intensity. Fluorescence intensity was then used to calculate the permeability coefficient (*P_e_*) or rate of passage through the monolayer:

*Pe* = *PS*/s, where *PS* (clearance) is the permeability surface area of the endothelial monolayer and *s* is the surface area of the filter (1.12 cm^2^).

*PS* is given by 1/*PS* = 1/*me* − 1/*mf*, where *me* and *mf* are the slopes of the curves corresponding to endothelial cells on filters and to filters only, respectively, with *me* and *mf* calculated by plotting the cleared volume against time.

The cleared volume was calculated by (*AUa* − *AUb*)/*Fi*, where *AUa* is the total fluorescence (arbitrary units) in the basal compartment, *AUb* is the background fluorescence, and *Fi* is the fluorescence of the initial solution (AU/mL).

### 4.5. mRNA and Protein Analysis

#### 4.5.1. RT-PCR

Any indicated treatments of the hCMEC/D3 monolayers were performed at confluence in 6-well culture plates. Following treatments, total RNA was extracted using the RNeasy Plus Mini procedure as described by the manufacturer (Qiagen, Valencia, CA, USA). RNA (500 ng) was used for cDNA synthesis using the SuperScript III First Strand Synthesis procedure as described by the manufacturer (Invitrogen Life Technologies, Carlsbad, CA, USA). Human intercellular adhesion molecule-1 (ICAM-1), vascular cell adhesion molecule-1 (VCAM-1), activated leukocyte cell adhesion molecule (ALCAM), β-actin, and GAPDH Taqman Primer/Probes were used for all Taqman Gene Expression Assays along with the Taqman Universal PCR Master Mix (Applied Biosystems, Foster City, CA, USA). Quantitative analyses of mRNA were conducted using an Applied Biosystems 7300 Real-Time PCR System (ThermoFisher Scientific, Waltham, MA, USA). Data were normalized using *C*t values for GAPDH and β-actin in each sample. Relative levels of mRNA expression were calculated as log_2_ relative units using the geometric mean of the average *C*t values for GAPDH and β-actin, and fold change was calculated for each treatment compared with untreated controls.

#### 4.5.2. Flow Cytometric Analysis

At confluence in 24-well culture plates, the indicated treatments of the hCMEC/D3 monolayers were performed. Following treatment, confluent hCMEC/D3 cells were harvested with trypsin. hCMEC/D3 cells were then stained with fluorescently conjugated antibodies specific for ICAM-1, VCAM-1, or ALCAM (eBioscience, San Diego, CA, USA) specific primary antibody followed by incubation with a fluorescently conjugated secondary antibody (eBioscience) in Staining Buffer (1× HBSS without Ca^2+^/Mg^2+^, FBS (3%), NaN_3_ (0.02%), and CaCl_2_ (2.5 mM)), then washed and fixed in 1% paraformaldehyde in 1× HBSS. Events were recorded using a FACSCalibur (BD Biosciences, San Jose, CA, USA), and the results were analyzed using FlowJo software (FlowJo, LLC, Ashland, OR, USA).

### 4.6. PBMC Adhesion Assay

At confluence in 24-well culture plates, the indicated treatments of the hCMEC/D3 monolayers were performed. Following termination of any indicated treatments of the monolayer, a suspension of 1 million PBMCs in co-culture media was added to the monolayer. The co-cultures were incubated for 30 min at 37 °C in CO_2_ (5%); unbound cells were removed through aspiration and washed twice with warmed media, followed by a final 1× HBSS rinse. All cells were then removed with trypsin and stained for flow cytometry. Staining for CD3 and CD14 (BD; eBioscience) allowed for distinguishing T-cell and monocyte populations from hCMEC/D3 cells. Adhered PBMCs were counted using the FACSCalibur, and the results were analyzed using FlowJo software.

### 4.7. Statistical Analysis

Because the raw expression values from FITC-D assay were right-skewed, they were log-transformed before the analysis. The mean values of each transformed expression were compared among untreated, morphine treated, and mannitol groups using the ANOVA method adjusting for possible time effects. The comparison of the gene expression from other assays were compared using the two-group *t*-test. In addition, *p*-values less than 0.05 were considered significant in all assays.

## Figures and Tables

**Table 1:** CAM (cellular adhesion molecule) surface expression correlates with CD3^+^ cell adhesion. Linear regression analysis based on results from three independent experiments performed in triplicate for both the cell adhesion assay and staining for CAM surface expression.

CAM Examined	Number of Expressors		MFI	
	*R*^2^	*p* Value	*R*^2^	*p* Value
ICAM-1	0.983	0.017	0.962	0.038
VCAM-1	0.998	0.002	−0.258	0.742
ALCAM	0.998	0.012	0.982	0.018

ALCAM, activated leukocyte cell adhesion molecule; ICAM-1, intercellular adhesion molecule-1; MFI, mean fluorescent intensity; VCAM-1, vascular cell adhesion molecule-1.
